# Assessing attitudes towards biostatistics education among medical students: adaptation and preliminary evaluation of the Chinese version survey of attitudes towards statistics (SATS-36)

**DOI:** 10.1186/s12909-024-05548-2

**Published:** 2024-06-06

**Authors:** Chen Li, Yuhai Zhang, Weidong Qin, Jielai Xia, Lei Shang, Ling Wang

**Affiliations:** Department of Health Statistics, School of Preventive Medicine, Ministry of Education Key Lab of Hazard Assessment and Control in Special Operational Environment, Fourth Military Medical University, No.169 Changlexilu Road, Xi’an, Shaanxi 710032 P. R. China

**Keywords:** Statistics education, Biostatistics, Attitudes, SATS, Factor analysis

## Abstract

**Background:**

Despite the numerous advantages of mastering biostatistics, medical students generally perceive biostatistics as a difficult and challenging subject and even experience anxiety during the courses. Evidence for the correlation between students’ academic achievements and their attitudes, indicating that attitudes at the beginning of the biostatistics course may affect cognitive competence at the end of the course and subsequently influence student academic performance. However, there are current disagreements regarding the measurement and evaluation of attitudes related to statistics. Thus, there is a need for standard instruments to assess them. This study was conducted to develop a Chinese version of the Survey of Attitudes Toward Statistics (SATS-36) in order to acquire a valid instrument to measure medical students’ attitudes toward biostatistics under Chinese medical educational background.

**Methods:**

The Chinese version SATS-36 was developed through translation and back-translation of the original scale, with subsequent revisions based on expert advice to ensure the most appropriate item content. The local adaption was performed with a cohort of 1709 Chinese-speaking medical undergraduate and graduate students enrolled in biostatistics courses. And then, the reliability, validity and discrimination of the questionnaires were evaluated through correlation coefficient calculation, factor analysis, parallel analysis and other methods.

**Results:**

The Chinese version SATS-36 consisted of 36 items and loaded a five-factor structure by factor analysis, which offered an alternative similar but not equal to that original six-factor structure. The cumulative variance contribution rate was 62.20%, the Cronbach’s α coefficient was 0.908, the Guttman split-half reliability coefficient was 0.905 and the test–retest reliability coefficient was 0.752. Discriminant analysis revealed small to large significant differences in the five attitude subscales.

**Conclusions:**

The Chinese version SATS-36 with good validity and reliability in this study can be used to evaluate the learning framework of Chinese medical students.

**Supplementary Information:**

The online version contains supplementary material available at 10.1186/s12909-024-05548-2.

## Background

Statistics has become increasingly important in most professions [1], especially in the field of biomedical sciences. Evidence-based medicine prompts the medical professionals to apply statistical tools for providing quality care, which requires an expert level of understanding the biostatistics for study design, data analysis, and result interpretation [2]. Thus, biostatistics, also known as medical statistics or health statistics in China, is increasingly taught as a required course of the medical curriculum across all categories in both developed and developing countries [3, 4].

Despite the advantages of mastering biostatistics, medical students generally perceive statistics as difficult and challenging subject and even experience anxiety or fear during statistics courses [4]. Anxiety about statistics is primarily attributed to poor mathematical background and logical thinking ability [5], or lack of research experience [6]. Numerous studies have reported that the negative perceptions towards biostatistics may affect the willingness, persistence and course achievement for medical students [6–9], and consequently hindering the development of students’ statistical thinking skills and application in clinical practice [10]. Therefore, students’ attitudes toward disciplines have garnered widespread attention in the education research literature [11]. Attitude toward statistics is commonly described as a multidimensional concept, which consists of affective (emotions and the motivation related to the classes and examinations), cognitive (beliefs and knowledge about the ability requested to learn statistics and about the discipline) and behavior (action tendencies in studying and the performance in examinations) components [12]. There are complex inter-relationships among various cognitive and non-cognitive factors that impact learning this subject. Students’ background in mathematics is considered to be the primary cognitive factor affecting their statistics achievements [13]. Non-cognitive factors such as students’ attitudes towards statistics also contribute to the understanding of statistical concepts and methods. While there is evidence for the relationships between achievements and students’ attitudes, here are disagreements in the measurement of attitudes [14]. Thus, acknowledged standard instruments are needed for their assessment.

At present, several inventories are used to assess attitudes towards statistics, such as the Statistics Attitude Scale [15], the Attitudes Toward Statistics(ATS) [16], the Survey of Attitude Toward Statistics(SATS) [17], and the Statistics Attitude Survey(SAS) [18]. Among them, the SATS-36 [19] and its predecessor SATS-28 [17] has been validated prudently and used the most widely to examine the psychometric properties across different populations, assess students’ statistics attitudes in response to course interventions and explore the relationships between students’ statistics attitudes and learning outcomes, and so on [11]. The SATS-28 [17] assesses four subscales (components) of attitude toward statistics: *Affect* (students’ positive and negative feelings about statistics); *Cognitiv*e *Competence* (students’ attitude about their intellectual knowledge and skills when applied to statistics); *Value* (attitude about the usefulness, relevance, and worth of statistics in personal and professional life); *Difficulty* (students’ attitude about the difficulty of statistics as a subject). Afterwards, two subscales, *Interest* and *Effort*, were added to the instrument and updated as SATS-36 [19]. The invariance of the measurement model and factor structure of SATS have been tested across gender and administration time. I Indicators related to each subscale and subscale covariances have been confirmed to be invariant [20, 21]. Strong correlations (from 0.92and 0.94) were also found between the six subscales [21]. Regarding reliability, the SATS showed a good internal consistency across samples. Specifically, Cronbach’s alpha coefficients value ranged from 0.80 to 0.89 for *Affect*, from 0.77 to 0.90 for *Cognitive Competence*, from 0.74 to 0.91 for *Value*, and from 0.64 to 0.86 for *Difficulty* [12, 17, 19]. Concerning validity, convergent validity was tested between the SATS scale and the other relating scales, with a substantial correspondence reported [12, 17]. Therefore, the psychometric properties of the instrument have been well documented and supported.

In view of this, the SATS has been translated and validated in many different languages in previously studies [13, 22–24]. As for China, some medical educational studies have also utilized SATS to investigate students’ attitudes toward statistics [25]. However, there is currently no standard Chinese version of SATS-36 available, which has been strictly developed and validated. Considering the diverse cultural contexts in China and the complex nature of medical educations, this research aims to investigate the psychometric properties of the Chinese version of the SATS-36 to acquire a valid instrument for measuring medical students’ attitudes toward biostatistics within Chinese-speaking medical educational context so as to support effective teaching reforms and intervention measures in the learning process.

## Methods

### Participants

This study has been conducted in accordance with the Declaration of Helsinki. The Ethics Committee of the Fourth Military Medical University carefully considered and approved the project proposal. All participants were informed and gave their consents for this research before they were investigated. Participants in the study were undergraduate and graduate students enrolled voluntarily in the mandatory biostatistics course during the 2020–2021 school year at the Fourth Military Medical University, China, which has a long history of imparting formal education in biostatistics. It is one of the earliest University to offer education in the field of biostatistics in China, which has an advanced teaching model as well as numerous achievements in teaching researches and teaching awards. These students come from different provinces across China and have diverse race, cultural and educational backgrounds, covering all medical categories, such as clinical medicine, stomatology, basic medicine, preventive medicine, pharmacy, and so on.

### Instruments

The original SATS-36 [19] contains 36 Likert-type items, which are grouped into six attitude subscales (components): *Affect* (6 items), *Cognitive Competence* (6 items), *Value* (9 items), *Difficulty* (7 items), *Interest* (4 items) and *Effort* (4 items). Responses for each item are ranked from 1 (strongly disagree) through 4 (neither disagree nor agree) to 7 (strongly agree), using the 7-point Likert method. Two forms of the SATS can be conducted. One form of the SATS is in the present tense, to be administered at the beginning of the course (pre-SATS), and the other one is in the future tense for the end of the course (post-SATS) [19]. According to the directions of the instrument, the scoring of the SATS-36 should be conducted as follows. Firstly, the responses of some negatively worded items should be reversed (response 1 is replaced by 7, 2 by 6, etc.) to ensure consistency for the measurement of all items, in which higher scores correspond to more positive attitudes. Then summing the item responses within each subscale and divided by the number of items. That is, the subscale scores are the means of the including items. And thus the subscale score still ranges from 1 to 7 with higher values indicating more positive attitudes [19, 26, 27].

**Fig. 1:**
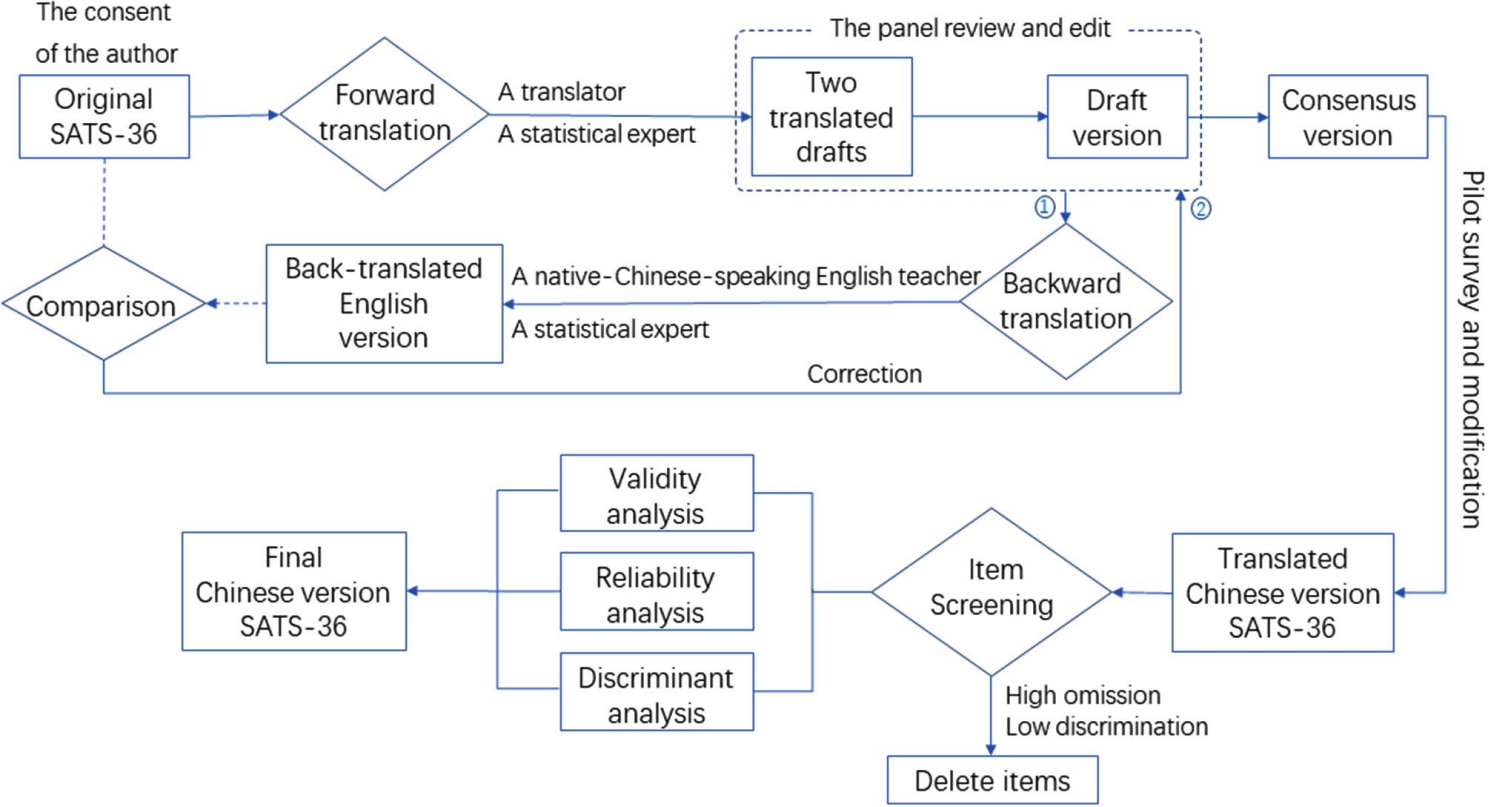
The flowchart of the translation and construction of Chinese version SATS-36

The process of the translation and construction of Chinese version SATS-36 was shown in Fig. 1. After obtaining consent from the author of the SATS-36 [19], our research group undertook the translation from English into Chinese. The SATS-36 adaptation was based on internationally accepted methodology for the cultural adaptation of questionnaires. In the first step, ‘‘forward translation’’ (translation of the original version from English to Chinese) was done to ensure the semantic and conceptual correspondence between the Chinese version and the original questionnaire. Translation was conducted by two independent professional translators, one specializing in statistics and the other a professional translator. After comparing the meanings and wordings of these two translated drafts, the most consistent translation items with the original version were selected for drafting the translated version of the SATS-36 following thorough panel discussions. The panel consisted of five experts in statistics education, the English language, and psychology. After review and editing by translators and experts, one single translation was formed. Subsequently, another stage of translation, known as ‘‘backward translation’’, involved translating the Chinese version of SATS-36 into English. A medical statistics professor who had lived and studied in United States for many years and a native-Chinese-speaking English teacher independently conducted back-translation for the drafted Chinese version, respectively. Discrepant items identified through comparison of the original and back-translated versions were reported to the panel for further discussion, and a second round of translation and correction was carried out as necessary to ensure consistency with the original items. Controversial items were discussed during the translation process, ultimately resulting in a consensus version of SATS-36 culturally adapted for Chinese students. The consensus version was pre-surveyed on 19 students, who were randomly selected from the participant population, to evaluate its understandability, acceptability and clarity. Following expert modification of the Chinese version based on feedback from the student participant, the final Chinese version of the SATS-36 was distributed in a validation study.

Participants in the validation study were also requested to provide demographic information with respect to age, gender, specialty, the background of logical thinking ability, mathematical basics, computer basics, and research experience.

### Procedures

The pre-SATS was conducted at the first introduction lesson of the biostatistics course. The purpose of the survey was briefly explained to the students, and they were informed that the participation was voluntary and that the results would remain anonymous. Then during the final week of the course, all participants were requested to complete the post-SATS of one’s own accord. The surveys were conducted individually in an in-class situation without discussion or collaboration. The students were assured that their responses would not impact their academic achievement or future learning process. Responses were collected with an online crowdsourcing free platform in China (called “Survey Star”, powered by www.wjx.cn), which provides functions equivalent to Amazon Mechanical Turk. Each survey took approximately 15 to 20 min to complete data collection.

### Statistical analysis

Raw data were checked for departures from normality and for the presence of outliers. The questionnaires with the same or blank responses exceeding 80% of the items were considered as invalid questionnaires. Descriptive statistics were calculated to determine students’ attitudes towards statistics. Continuous variables were expressed by mean ± standard deviation(SD) when the data was approximately normally distributed, otherwise median and quartile were used instead. The categorical variables were expressed by numbers and percentages. Dispersion tendency analysis, factor analysis and reliability tests were used for item analysis. Items were screened using standard deviations, factor loadings of item scores and Cronbach’s α coefficient. Item deletion was considered when the standard deviation of the item score < 0.85, the factor loading < 0.4 and the Cronbach’s α coefficient of the whole scale was greater after the removal of the item than before its removal. The coefficient of correlation between a subscale score and the total score can be used to evaluate the content validity of a questionnaire. Exploratory factor analysis (EFA) was conducted using the largest variation method and orthogonal rotation and the number of extraction factors was determined by parallel analysis when the actual eigenvalue of the data in the scree plot curve falls below the average eigenvalue of the curve of the random matrix. Confirmatory factor analysis (CFA) of the extraction factor model was performed using data random samples from the participants. With these methods, we explored the factor structure and tested the scale’s construct validity.

*t* tests and the mixed effects model were used to compare SATS-36 scores and the subscale score means among subjects with different characteristics, thereby examining the scale’s discriminant analysis. Ordinal coefficient α [28] and Cronbach’s α coefficients [29] were calculated to evaluate internal consistency, with higher values indicating good reliability. The test–retest reliability coefficient was used to evaluate scale stability, and the Guttman split-half reliability coefficients are used to evaluate equivalence. Analyses were performed using SAS 9.4 (SAS Institute Inc, USA), SPSS23.0 (IBM SPSS Statistics) and Mplus 6.0 (LindaMuthen, BengtMuthen).

Sometimes, the Likert type data are used in questionnaire response reflecting attitudes or levels of cognition. Likert-type data are ordinal data and analyzing ordinal data improperly as quantitative data may lead to systematic errors, such as Type I errors, loss of power and even inversions of effect estimation [30]. The graded response models in an item-response theory (IRT) framework are suggested for the ordinary data in the scale validation [31, 32]. For this study, the original SATS-36 using the 7-point Likert method, are developed for the scoring, analysis and evaluation as quantitative data. We also explored IRT analysis treating 7-point Likert response as ordinary data to validate accuracy and robustness of this translation version. Since the subscale scores were calculated by the means of the including items predefined in the development of original SATS-36 scale, we only conducted graded response models analysis for each single item. The results confirmed that the items exhibited moderate to high discrimination totally. The item characteristic curve (ICC) analysis showed relatively high predicted probability of a certain response, which meant acceptable discriminant. And the item information curve (IIC) analysis suggested that the items provided significant contributions for the measure of the latent subscale. The empirical reliability and marginal reliability were calculated as 0.9486 and 0.9503, respectively, which was considered sufficiently to indicate the item reliability. We considered IRT analysis treating 7-point Likert response as ordinary data indicated similar accuracy and robustness (the detail results can be found in supplementary materials). Therefore, despite these risks, we treated our data as metric in order to compare our results with the extant literature.

## Results

### Participants characteristics

Of 1733 questionnaire distributed, 1721 questionnaires were collected and 1709 questionnaires were valid (valid call-back rate, 98.62%). For the 24 invalid questionnaires, nine were blank and 15 had the same responses across more than 80% of all the items. For the 1709 valid questionnaires, 1093 students completed the pre-SATS surveys and 1503 students completed the post-SATS surveys. Among these participants, 1070 are undergraduate and 639 are graduate. More participants were male (53.89%). The mean ages of the students were 20.71 ± 1.65 years (range 18–23 years) for undergraduates and 25.55 ± 3.62 years (range 21–38 years) for postgraduates. Most of students were majoring in clinical medicine (42.12%), basic medicine (11.51%) and stomatology (7.25%). Participants reported good ability in logical thinking (4.61 ± 1.10), but not confident enough on their mathematical ability (4.10 ± 1.25) and computer skills (3.80 ± 1.27). The general characteristics of participants are shown in Table 1.

**Table 1:** Main characteristics of the participants

Characteristics	Categories	Pre-SATS survey *n* (%)				Post-SATS survey *n* (%)			All participants *n* (%)		
		Undergraduates 769(70.36%)		Graduates 324(29.64%)		Undergraduates 856(56.95)	Graduates 647(43.05%)		Undergraduates 1070(62.60)	Graduates 639(37.40)	Total 1709(%)
Age (mean ± SD)		20.39 ± 2.20		25.20 ± 4.20		20.98 ± 0.79	25.72 ± 3.27		20.71 ± 1.65	25.55 ± 3.62	22.52 ± 3.48
Gender	Male	454(59.04)		154(47.53)		506(59.11)	312(48.22)		632(59.08)	307(47.99)	939(54.94)
	Female	315(40.96)		170(52.47)		350(40.89)	335(51.78)		438(40.92)	332(52.01)	770(45.06)
Logical thinking ability	Poor	199(25.93)		35(10.80)		92(10.75)	28( 4.33)		144(13.49)	41( 6.49)	186(10.87)
	Neutral	423(55.03)		187(57.72)		237(27.69)	233(36.01)		349(32.63)	276(43.25)	626(36.60)
	Good	146(19.05)		102(31.48)		527(61.57)	386(59.66)		577(53.88)	321(50.26)	898(52.53)
Mathematics background	Poor	312(40.57)		108(33.33)		156(18.22)	78(12.06)		308(28.80)	122(19.16)	431(25.20)
	Neutral	329(42.78)		168(51.85)		283(33.06)	293(45.29)		403(37.66)	303(47.48)	706(41.33)
	Good	128(16.64)		48(14.81)		417(48.71)	276(42.66)		359(33.54)	213(33.37)	572(33.48)
Computer background	Poor	419(54.49)		122(37.65)		254(29.67)	102(15.77)		443(41.42)	147(23.07)	591(34.56)
	Neutral	266(34.59)		167(51.54)		295(34.46)	325(50.23)		369(34.52)	324(50.67)	693(40.56)
	Good	84(10.92)		35(10.80)		307(35.86)	220(34.00)		257(24.06)	168(26.26)	425(24.88)

### Item screening

To avoid misunderstanding or decreasing of precision in responses, items with high omission rates (> 5%) and low discrimination (standard deviation of the item score < 0.85) would be removed with a prudential panel discussion. The standard deviations for all the 36 items were higher than one and factor loadings for all items were > 0.4 for both the pre and post versions. The results showed that no item satisfied the exclusion criteria. For the Cronbach’s α coefficients of 0.908 and 0.894 for the pre-SATS and post-SATS, the removal of any SAT-36 item could decrease the Cronbach’s α coefficient for the scale.

To ensure that the constructs have not changed in the translation, we also assessed the normality of data within the original subscales. Following the parcelling procedure referenced by the previous reported literatures [17, 21, 22], items within each original subscale of SATS-36 [19] were grouped into parcels, and univariate distributions of parcels were examined for assessment of normality. This procedure could help avoid the inherent non-normality associated with single item distributions [33]. All these indices attested that the departures from normality were acceptable. Thus, these 36 items were deemed suitable for inclusion in the translated Chinese version of the SATS-36 (Table 2).

**Table 2:** Descriptive statistics for items parcels according to the original six subscales of SATS-36

	Pre-SATS					Post-SATS			
Item	Mean	SD	Factor loading	Cronbach’s α coefficient after item deletion		Mean	SD	Factor loading	Cronbach’s α coefficient after item deletion
**Affect**	4.543	1.173				4.475	1.180		
q3	5.264	1.439	0.699	0.883		5.412	1.471	0.712	0.878
q4	4.140	1.711	0.605	0.883		3.791	1.753	0.596	0.876
q15	4.096	1.753	0.596	0.884		4.073	1.780	0.614	0.873
q18	4.005	1.703	0.632	0.882		3.644	1.761	0.618	0.874
q19	4.914	1.439	0.747	0.882		5.050	1.452	0.772	0.878
q28	4.833	1.718	0.686	0.881		4.821	1.791	0.693	0.870
**Cognitive Competence**	4.523	0.936				4.434	0.930		
q5	3.789	1.502	0.441	0.888		3.314	1.501	0.408	0.881
q11	5.597	1.462	0.556	0.883		5.708	1.501	0.597	0.873
q26	3.435	1.561	0.518	0.884		3.606	1.585	0.482	0.878
q31	5.377	1.330	0.655	0.884		5.345	1.386	0.732	0.878
q32	4.863	1.360	0.672	0.885		4.746	1.467	0.635	0.879
q35	4.079	1.600	0.566	0.885		3.888	1.693	0.574	0.873
**Value**	5.538	0.996				5.730	1.040		
q7	6.403	1.141	0.502	0.896		6.372	1.272	0.582	0.875
q9	5.671	1.341	0.638	0.893		5.978	1.281	0.685	0.881
q10	5.715	1.329	0.624	0.886		5.872	1.335	0.642	0.881
q13	5.461	1.586	0.508	0.884		5.680	1.621	0.605	0.876
q16	5.289	1.505	0.592	0.886		5.517	1.592	0.700	0.872
q17	4.781	1.542	0.463	0.885		5.000	1.620	0.543	0.880
q21	5.120	1.519	0.526	0.887		5.269	1.617	0.599	0.874
q25	5.643	1.413	0.624	0.885		5.920	1.389	0.705	0.874
q33	5.758	1.394	0.672	0.885		5.963	1.390	0.723	0.873
**Difficulty**	2.838	0.789				2.620	0.730		
q6	3.833	1.459	0.641	0.893		3.754	1.516	0.412	0.883
q8	2.672	1.478	0.405	0.892		2.263	1.366	0.461	0.880
q22	3.344	1.498	0.567	0.882		3.249	1.601	0.520	0.884
q24	2.309	1.233	0.562	0.884		1.955	1.129	0.623	0.885
q30	2.590	1.405	0.536	0.886		2.615	1.514	0.541	0.884
q34	2.445	1.306	0.535	0.883		2.082	1.249	0.633	0.884
q36	2.672	1.348	0.433	0.884		2.422	1.373	0.481	0.885
**Interest**	4.840	1.296				5.104	1.260		
q12	4.457	1.539	0.616	0.886		4.882	1.577	0.653	0.875
q20	5.023	1.441	0.783	0.882		5.227	1.435	0.754	0.878
q23	4.839	1.452	0.755	0.882		5.059	1.463	0.731	0.878
q29	5.041	1.534	0.732	0.889		5.248	1.542	0.749	0.878
**Effort**	6.313	0.911				6.343	0.730		
q1	6.412	1.080	0.756	0.886		6.633	0.948	0.772	0.884
q2	6.340	1.051	0.768	0.887		6.303	1.085	0.746	0.883
q14	5.961	1.253	0.614	0.884		5.783	1.408	0.459	0.876
q27	6.540	1.079	0.581	0.883		6.651	1.010	0.640	0.884

### Validity

#### Content validity

All coefficients of correlation between the original subscales and total scale (0.43–0.87) were greater than coefficients of correlation between subscale scores (0.23–0.78). Specifically, the “Difficulty” subscale had the weakest correlation with the total scale with the correlation coefficient of 0.43. Following that, the “Effort” subscale showed a little stronger correlation coefficient of 0.65. The correlation coefficients for the other subscales were all above 0.80, of which the “Affect” subscale showed the highest coefficients of 0.87. As for the correlations between subscales, the correlation between “Interest” and “Difficulty” subscales was the weakest, while the correlation between “Affect” and “Cognitive Competence” was the strongest. The correlations between other subscales ranged from 0.45 to 0.73, which showed moderate correlations.

#### Construct validity

The KMO and Bartlett’s test showed that the data was suitable for factor analysis (Kaiser–Meyer–Olkin value = 0.93, Bartlett’s spherical test value = 54442.67, concomitant probability < 0.001). Thus, an exploratory factor analysis (EFA) was performed on the post-SATS. To determine the number of factors to extract, a parallel analysis was performed. The parallel analysis generated data for 20 random samples and then performed EFA on each of the 20 data sets, recording each of the eigenvalues. The EFA results showed that five factors were extracted with an orthogonal rotation, which was also supported by the inspection of the parallel analysis and the eigenvalues from the factor analysis on the SATS items. The total variance explained equals 62.20%. The eigenvalues of the five factors were 7.68, 5.00, 3.89, 3.15 and 2.67, and the variance contribution rates were 21.33%, 13.90%, 10.81%, 8.75% and 7.41%.

Based on the implied meanings of the items with the greatest loadings, the five factors were deemed to have identical factor interpretations (i.e., all items had the strongest coefficients in the same factor in these rotated matrices) with the pattern matrix generating the most interpretable simple structure as shown in Table 3. The first factor reflected students’ interest and positive expectation towards this course. It included all the items from the original “Interest” subscale, two items from the “Affect” subscale (Item 3 and 19), two items from the “Value” subscale (Item 9 and 17), one item from the “Cognitive Competence” (Item 32) and one items from the “Difficulty” subscale (Item 22) [19]. Students’ interest towards biostatistics was not only rooted in the emotion, but in a comprehensive consideration of the cognitive, value and difficulty of the course. It was precisely because students approved the worth and importance of statistics and enjoyed the learning process, they generated the positive and strong sense of identification with the subject. Based on it, students are willing to learn biostatistics. Therefore, we called this factor as *Willingness* subscale. The second factor included all the items but two (Item 9 and 17) from the original “Value” subscale so we retained this name. The third factor were very similar to the original “Affect” subscale excluding Item 3 and 19. However, the remaining items were not just the feelings towards this course but some negative emotional states concerning statistics. There may be some stress, fear, nervous and even frustration for students’ attitudes towards this course, which was contrast to the first factor. Thus, we concluded them as *Pressure* subscale. The fourth factor was completely consistent with the original “Effort” subscale, which reflected the effort and time students expending to learn statistics. The fifth factor included most of items from the original “Difficulty” subscale except for Item 4 and 22. Students still considered that statistics to be a complicated subject and they have to adopt a new way of thinking to study statistics. Thus, the Chinese SATS-36 versions retained three original subscales of *Value*, *Effort* and *Difficulty* and loaded two new subscales of *Willingness* and *Pressure* .

**Table 3:** Factor matrix for the Chinese version SATS-36

Factor name and item		Original subscale*	Loading
*Willingness*			
q19	I will enjoy taking statistics courses.	A	0.831
q29	I am interested in learning statistics.	I	0.806
q23	I am interested in understanding statistical information.	I	0.806
q20	I am interested in using statistics.	I	0.802
q12	I am interested in being able to communicate statistical information to others.	I	0.773
q3	I will like statistics.	A	0.762
q31	I can learn statistics.	C	0.751
q32	I will understand statistics equations.	C	0.724
q17	I use statistics in my everyday life.	V	0.671
q22	Statistics is a subject quickly learned by most people.	D	0.608
q10	Statistical skills will make me more employable.	V	0.574
q6	Statistics formulas are easy to understand.	D	0.570
q9	Statistics should be a required part of my professional training.	V	0.559
***Value***			
q25	I will have no application for statistics in my profession.	V	0.824
q33	Statistics is irrelevant in my life.	V	0.814
q16	Statistical thinking is not applicable in my life outside my job.	V	0.771
q13	Statistics is not useful to the typical professional.	V	0.762
q21	Statistics conclusions are rarely presented in everyday life.	V	0.731
q7	Statistics is worthless.	V	0.721
q11	I will have no idea of what’s going on in this statistics course.	C	0.569
***Pressure***			
q4	I will feel insecure when I have to do statistics problems.	A	0.744
q18	I will be under stress during statistics class.	A	0.726
q15	I will get frustrated going over statistics tests in class.	A	0.708
q28	I am scared by statistics.	A	0.668
q35	I will find it difficult to understand statistical concepts.	C	0.579
q5	I will have trouble understanding statistics because of how I think.	C	0.569
***Effort***			
q1	I plan to complete all of my statistics assignments.	E	0.832
q2	I plan to work hard in my statistics course.	E	0.751
q27	I plan to attend every statistics class session.	E	0.666
q14	I plan to study hard for every statistics test.	E	0.517
***Difficulty***			
q30	Statistics involves massive computations.	D	0.705
q34	Statistics is highly technical.	D	0.664
q36	Most people have to learn a new way of thinking to do statistics.	D	0.558
q24	Learning statistics requires a great deal of discipline.	D	0.541
q26	I will make a lot of math errors in statistics.	C	0.478
q8	Statistics is a complicated subject.	D	0.465

*: The original subscale of A: Affect; C: Cognitive Competence; V: Value; D: Difficulty; I: Interest; E: Effort

The inter-relationships among the subscale components were all statistically significant, except between *Difficulty* and *Value*. The *Willingness* and the *Effort* subscales were strongly related to each other (*r* = 0.563), as well as the *Difficulty* and *Effort* subscales (*r* = -0.581). The *Value* and *Difficulty* subscales were moderately related to the *Pressure* subscale positively. Besides, *Effort* subscale was negatively correlated with *Pressure* and *Difficulty* subscales, as well as the *Willingness* with *Difficulty* subscales (Table 4).

**Table 4:** Correlations among the SATS subscale scores

	Willingness	Value	Pressure	Effort
**Value**	0.321^*^			
**Pressure**	0.236^*^	0.514^*^		
**Effort**	0.563^*^	0.185^*^	-0.111^*^	
**Difficulty**	-0.337^*^	0.039	0.425^*^	-0.581^*^

*: The correlation was statistically significant (*P* < 0.05)

#### Cross-validation of the factorial structure

In order to cross-validate the subscale structures, we selected half of the questionnaires randomly to ensure the constructs unchanged in the translation and to reduce the risk of the model being driven by chance factors associated with specific sample characteristics. The five factor structures obtained by the EFA were tested on the data from the calibration sample with confirmatory factor analysis (CFA). The structural validity of questionnaires was found to be adequate (${x}_{584}^{2}=1690.332, p<0.0001$) with the degree of freedom ratio 2.89(< 3.0), the comparative fit index (CFI) 0.837(> 0.80), Root Mean Square Error Of Approximation (RMSEA)0.071(< 0.08) and Standardized Root Mean Square Residual (SRMR) was (0.091) < 0.1.

**Fig. 2:**
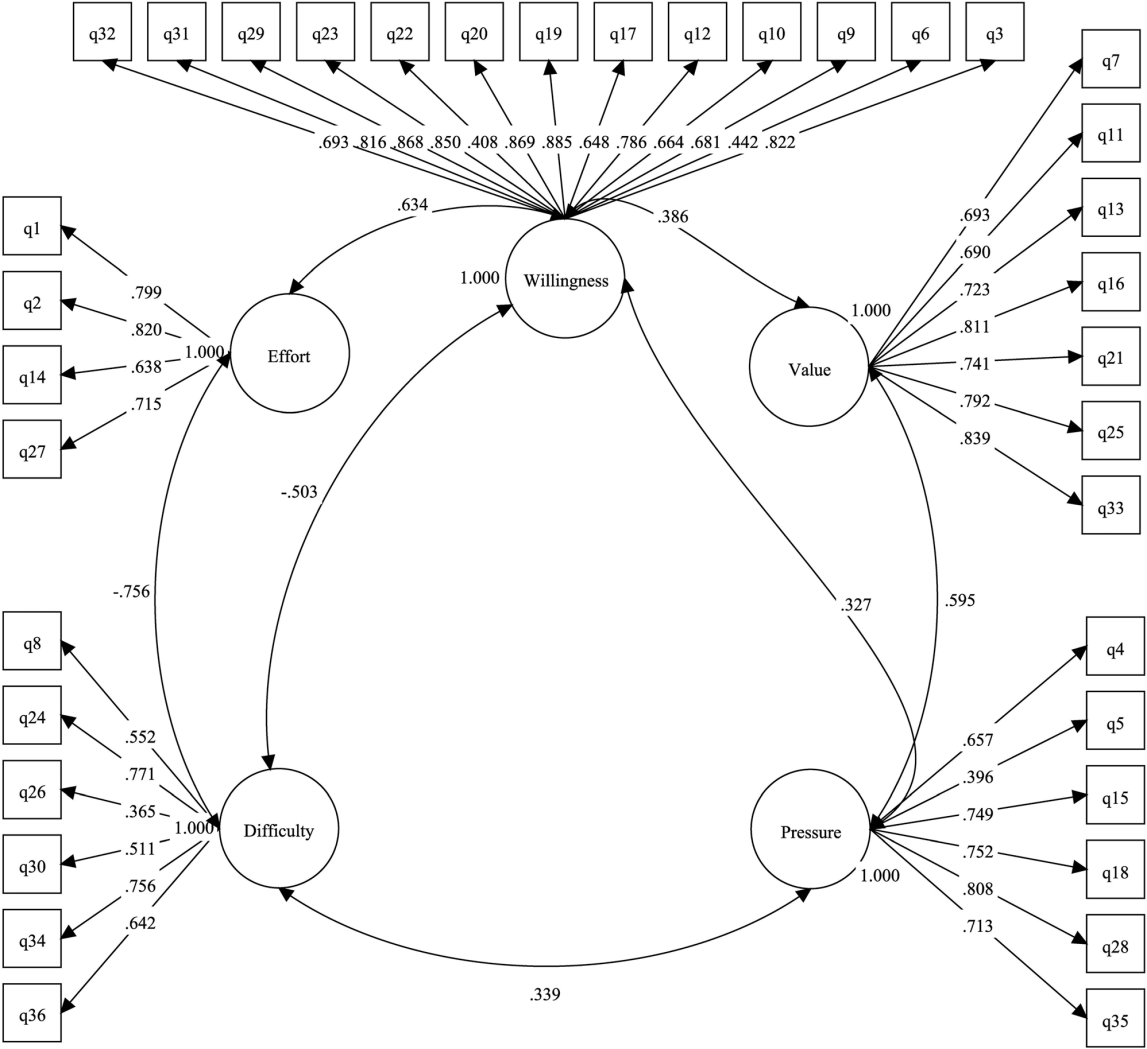
Path diagram for the five-factor model of Chinese version SATS-36

These results were detailed in Fig. 2. As indicated, the *Willingness*, *Value* and *Effort* subscales were positively related to one another (standardized regression coefficient *β* were 0.386, 0.212 and 0.634 respectively). The *Value* and *Willingness* also positive correlated with *Pressure* moderately (*β* = 0.595 and *β* = 0.595). While, *Pressure* correlated weakly with *Effort* (*β*=-0.066). And *Difficulty* was negatively related to *Willingness*, *Value* and *Effort* subscales (*β*=-0.503, *β*=-0.084 and *β*=-0.756).

### Reliability

For surveys, reliability is usually regarded as the internal consistency of the items within each scale, which reflects the degree of interrelationship among students’ responses to the scale’s items. Although Cronbach’s α coefficient [29] is commonly used in unidimension test score reliability assessment, there are always misuse or overuse for most multi-dimension scales [34]. As researchers’ discussion, Cronbach’s α coefficient has limited usefulness for Likert type rating response scale, because it assumes that the scale is unidimensional with the item responses as continuous data [35]. In this study, we calculated the ordinal coefficient α for reliability assessment of the Likert response data following Zumbo’s methods [28]. And also, Cronbach’s α coefficient was provided for the comparison with the original SATS and other relating researches. The results showed that revealed that the ordinal coefficient α was slightly larger than Cronbach’s α, which was found to be more precise and closer to the theoretical value by the simulation study in Zumbo’s research. In this study, the ordinal coefficient α were 0.901 and 0.887 for all the 36 items in the pre-SATS and post-SATS respectively, indicating good stability as shown in Table 5. The reliability coefficients for the subscales ranged from 0.725 to 0.937. In previous studies, the range of Cronbach’s α values for subscales includes: the original “Affect” from 0.80 to 0.89, “Value” from 0.74 to 0.90, “Difficulty” from 0.64 to 0.81 [19]. Our reliability coefficients from EFA were similar to these estimations [19]. As for the *Willingness* and *Pressure* subscales we detected in this study, the Ordinal coefficients α coefficients reaches 0.937 and 0.850 and Cronbach’s α coefficients of 0.935 and 0.842, which were higher than those in the “Interest” and “Affect” subscales from the original dimension. The *Difficulty* subscale tended to exhibit the lowest level of internal consistency, but it was considered at least adequate. Thus, we considered that these ordinal coefficients α coefficients were sufficiently high to indicate scale reliability.

We also verify the split-half reliability and retest reliability as shown in Table 5. All the items were listed in order of their item numbers. The Guttman split-half coefficients showed that the total SATS-36 scale and its subscales had good split-half reliability with the range from 0.74 to 0.92. For the retest reliability, we randomly sampled 100 participants from undergraduates and graduates respectively and retested the scale within 3 weeks. The retest reliability coefficients were considered at least adequate to indicate the retest reliability.

**Table 5:** Reliability coefficients of SATS with subscales

Measure	Cronbach’s α			Ordinal coefficients α		Guttman split-half coefficient	Retest reliability coefficient
	Pre-SATS	Post-SATS		Pre-SATS	Post-SATS		
SATS total	0.908	0.894		0.901	0.887	0.905	0.752
Willingness	0.917	0.935		0.919	0.937	0.916	0.757
Value	0.861	0.901		0.863	0.902	0.847	0.743
Pressure	0.842	0.842		0.849	0.850	0.817	0.725
Effort	0.831	0.809		0.834	0.808	0.798	0.730
Difficulty	0.706	0.758		0.725	0.756	0.738	0.710

### Discriminant analysis

We explored the discriminant validity associated with some participants’ basic characteristics. Taking the means of item responses in each subscale as the subscale score, the mixed effects model was used for the mean comparisons between these characteristics’ categories. The *Willingness* and *Pressure* subscale scores differed significantly according to students’ gender, education level, logical thinking ability, mathematical basic and computer basics (all *P* < 0.01). This study found that the female tended to have lower scores on the *Willingness* and *Pressure* subscales, which was consistent with the established or verified invariance of factor structure on gender in some literatures [36, 37]. Moreover, the *Value* of *Effort* subscale score differed significantly across different education level, logical thinking ability, mathematical basic and computer skills (all *P* < 0.01). Graduate students, perhaps due to their greater research experience and positive expectations on biostatistics, attained higher subscale scores compared to undergraduate students. Similarly, students with proficient logical thinking, mathematics, and computer skills demonstrated more positive attitudes than those with weaker foundations. However, there were almost no differences in *Difficulty* scores across any of the subject characteristics (Table 6). It is worth noting that it should be caution taking means of item responses as the subscale score for ordinal data in this section. Therefore, we also conducted the discrimination assessment for each single item treating Likert response as ordinary data with item-response theory (IRT) analysis, which was shown as table s1 and figure s1 in the supplement material.

**Table 6:** Discriminant validity of the subscale scores according to participant characteristics

Factor	Categories	Willingness	Value	Pressure	Effort	Difficulty
Total		5.01 ± 1.02	5.76 ± 1.15	3.90 ± 1.23	6.38 ± 0.73	2.46 ± 0.80
Gender	Male	5.13 ± 1.12	5.75 ± 1.24	4.02 ± 1.28	6.32 ± 0.92	2.49 ± 0.93
	Female	4.82 ± 1.06	5.81 ± 1.10	3.81 ± 1.27	6.37 ± 0.87	2.49 ± 0.92
		< 0.01	0.30	< 0.01	0.21	0.99
Population	Undergraduates	4.80 ± 1.20	5.56 ± 1.25	3.93 ± 1.34	6.15 ± 1.04	2.62 ± 1.02
	Graduates	5.22 ± 0.90	6.00 ± 1.03	4.00 ± 1.21	6.61 ± 0.58	2.31 ± 0.76
		< 0.01	< 0.01	0.65	< 0.01	< 0.01
Logical thinking ability	Poor	3.68 ± 1.33	4.87 ± 1.40	2.71 ± 1.34	5.99 ± 1.32	2.33 ± 1.34
	Neutral	4.59 ± 0.91	5.54 ± 1.12	3.56 ± 1.06	6.20 ± 0.93	2.47 ± 0.89
	Good	5.36 ± 0.96	6.02 ± 1.09	4.27 ± 1.23	6.47 ± 0.78	2.52 ± 0.87
		< 0.01	< 0.01	< 0.01	< 0.01	0.86
Mathematics background	Poor	4.10 ± 1.22	5.28 ± 1.25	3.15 ± 1.27	6.12 ± 1.09	2.34 ± 1.08
	Neutral	4.76 ± 0.95	5.69 ± 1.10	3.70 ± 1.14	6.28 ± 0.91	2.51 ± 0.92
	Good	5.47 ± 0.93	6.02 ± 1.18	4.37 ± 1.23	6.47 ± 0.79	2.52 ± 0.87
		< 0.01	< 0.01	< 0.01	< 0.01	0.31
Computer background	Poor	4.38 ± 1.30	5.46 ± 1.31	3.44 ± 1.38	6.13 ± 1.24	2.44 ± 1.20
	Neutral	4.87 ± 0.87	5.78 ± 1.04	3.85 ± 1.10	6.35 ± 0.74	2.51 ± 0.81
	Good	5.53 ± 0.94	5.99 ± 1.19	4.34 ± 1.28	6.48 ± 0.76	2.52 ± 0.84
		< 0.01	< 0.01	< 0.01	< 0.01	0.56
Survey time	Pre-SATS	4.76 ± 0.96	5.51 ± 1.04	3.67 ± 1.15	6.35 ± 0.80	2.52 ± 0.81
	Post-SATS	4.73 ± 0.90	5.65 ± 1.02	3.66 ± 1.19	6.21 ± 0.68	2.54 ± 0.77
		0.77	0.18	0.94	0.07	0.76

## Discussion

Attitudes at the beginning of the biostatistics course may affect cognitive competence at the end of the course and subsequently influence student academic performance. This suggested the importance of positively changing not only students’ cognitive competency but also their perception and achievement in acquiring cognitive competency during the biostatistics course [6, 25]. In this study, a Chinese version of the well-known instrument SATS-36 was developed, and validated to measure Chinese-speaking medical students’ attitudes towards biostatistics.

The translation of the Chinese version SATS-36 was established through a cross-validation procedure, means and standard deviations of the SATS-36 original subscales were comparable to previous studies [19, 22, 24]. Generally, the SATS-36 original subscales’ means were above neutral attitude, especially *Value* and *Effort* subscales, implying positive attitudes towards statistics. While, medical students hold a more negative attitudes on the original *Difficulty* subscale compared to the other researches. Most of Chinese medical students considered that biostatistics was difficult but willing to pay full attention and efforts to learn it. VanHoof et al. had deleted several *Difficulty* items (Item 22, 34, and 36) due to low factor loadings [38]. He suggested deleting item 22 because this item might pertain to how most people perceive statistics, whereas other items focus more on students’ attitudes towards statistics. In Hommik’s study, five *Difficulty* items were deleted in total (Item 6, 22, 24, 30 and 34). Since it surveyed secondary school students in Estonia, who might not distinguish statistics from mathematics generally, at least when it came to formulas and calculating. In this study, no item was deleted in the item screening process. The standard deviations for all the 36 items were higher than one and factor loadings for all items were > 0.4 for both the pre and post versions. All the Cronbach’s α coefficients of the whole scale were not greater after the removal of any item than before its removal. Thus, all 36 items were appropriate and screening into the Chinese version of the SATS-36.

For the construct validity, the original investigator used CFA to support a four-factor structure of SATS-28 and a six-factor structure of SATS-36 [19]. In the more recent studies, researches suggested that it might be an instrument with only two components [12] or three components [39] of SATS-28 and seven components of SATS-36 [23] for the adaption of different language samples. In this study, the results of EFA showed that a five-factor solution for the Chinese version SATS-36offers an alternative that is similar but not identical to the original six-factor structure. In addition to cultural differences, the participants’ age, educational level and scientific experience might also have effects on medical students’ perceives. All five subscales loaded strongly and significantly and the goodness of fit indices of CFA had been verified. In more detail, the *Willingness* subscale was a comprehensive consideration of interest, course value, cognition and difficulty, which loaded all the items of the original “Interest” subscale and some other subscales. Students perceiving importance of biostatistics and approving its value could generate a positive interest and willingness on the subject, and leading to an enjoyable learning process. It might be challenging for students to distinguish between the emotion impact of course and their interest, or the perception of course value and their cognitive competence. These discoveries align with previous findings from validation studies in other languages, in which the original Affective and Cognitive competence subscales loaded onto a single factor. Thus, we considered the *Willingness* subscale as a comprehensive measure of medical students’ attitudes toward statistics course. Another new subscale constructed in this version was *Pressure* subscale, which essentially included all the items from the original “Affect” subscale expect Item 3 and 19. The remaining items represented various negative emotional states concerning statistics, such as stress, fear, nervous and even frustration towards this course. Thus, we renamed as “*Pressure*” subscale.

Internal consistency coefficients were also in accordance with other validation studies [12, 19, 22, 23] and supported the reliability of each subscale with the Cronbach’s alpha of 0.91 for the overall scale. Scores of the *Value* and *Effort* subscales were by and large consistent with previous research [12, 13] even if in the Chinese sample estimates were lower on the *Difficulty* scale.

Concerning the Discrimination, almost all the subscale scores apart from *Difficulty* showed significant difference in different gender, education level, logical thinking ability, mathematical basic and computer skills. In our study, female students tended to score lower on the *Willingness* and *Pressure* compared to the male students, which was similar to some previous researches [21, 39, 40]. We also found that self-rating of ability in mathematics was a factor influencing statistics attitudes, which was consistent with previous studies [41, 42]. Hannigan [40] reported that the strongest predictor of most of the attitude components was how well medical students felt they had performed in mathematics in the past.

It is worth noting that the original SATS-36 are developed for the scoring, analysis and evaluation as quantitative data. The attitude subscales are calculated by the means of the including items after reversing some negatively worded items. As far as we know, the localization SATS scales were also adapted and validated as quantitative data [13, 22–24]. Therefore, we conducted the scale validation similar to the original SATS and previous studies, such as descriptive statistics and CFA. However, we also explored IRT analysis treating 7-point Likert response as ordinary data to validate its accuracy and robustness, in which the results showed an acceptable discrimination and reliability. Although it was an innovative attempt and might not fully align with the original intention and development of SATS-36, we still emphasize the importance of applying the correct analysis methods for the ordinary data to control the systematic errors [30, 31]. Analyzing ordinal data improperly as metric may systematically lead to Type I errors, loss of power and even inversions of effects [30]. The graded response models in an item-response theory framework may be more suitable for the ordinary data in the scale validation [31].

This study had three highlights. First, although SATS-36 has been applied in some Chinese medical teaching researches, there was still no standard Chinese version of SATS-36 available. This study provided the first adaptation to investigate the psychometric properties of the Chinese version SATS-36 with a rigorous process of development and validation, which can be widely applied to the exploration of biostatistics teaching in China and provide support in terms of measurement scales. Second, the participants in the study included almost two thousand medical undergraduate and graduate students with diverse cultural, educational backgrounds and medical categories. They received biostatistics education from the university with a long history and good reputation in China, in which the large sample have a good extrapolation. Third, this study loaded a five-factor structure by factor analysis, which offered an alternative similar but not entirely equivalent to the original six-factor structure. We consider the local adaption has good validity and reliability, which can be used to evaluate the learning framework of Chinese-speaking medical students.

The main limitation of the present study is that we could not evaluate the criterion validity of the scale because there were no other tools available for the evaluation of students’ attitudes toward statistics in China. Additionally, the participants were recruited from a medical university. Considering the diversity in teaching modes and methods of biostatistics across different universities, this can inevitably impact students’ attitudes towards biostatistics. In the future, we will expand the test area and participant variety to explore the predictability of the medical students’ attitudes towards biostatistics on their course achievements.

## Conclusion

The present study provided evidence for the appropriate metric properties of the Chinese version of SATS-36. Exploratory factor analysis detected a five-factor structure of the scale. Good indices for both validity and reliability were obtained. The results reconfirmed the psychometric characteristics of SATS scale observed in medical student populations. This Chinese version SATS-36 might be a reliable and a valid instrument for identifying medical student attitudes towards biostatistics in the Chinese medical education, which could support future researches on the relationship between perception towards statistics and course achievements in China.

### Electronic supplementary material

Below is the link to the electronic supplementary material.

Supplementary Material 1

## Data Availability

The datasets used and/or analyzed during the current study available from the corresponding author on reasonable request.
