# Supplementary material for: Assessing attitudes towards biostatistics education among medical students: adaptation and preliminary evaluation of the Chinese version survey of attitudes towards statistics (SATS-36)
Source: BMC Med Educ. 2024 Jun 6;24:634. doi: 10.1186/s12909-024-05548-2 (PMC11157741; doi:10.1186/s12909-024-05548-2)
Supplement: Supplementary file 1 — Supplementary Material 1 [file 12909_2024_5548_MOESM1_ESM.docx]

**Supplementary materials**

We explored item-response theory (IRT) analysis treating 7-point Likert response as ordinary data to validate accuracy and robustness of our translation version. Since the subscale scores were calculated by the means of the including items predefined in the development of original SATS-36 scale, we only conducted graded response models analysis for each single item.

Table s1 showed the items’ discriminations with the item difficulties in the six boundaries in the 7-point Likert item expressed as b1-b6. The results showed that the items exhibit good discrimination totally. There are 24 items with discrimination values exceeding 1, among which the discriminations of items q3, q19, q23, q29 and q31 exceeding 2, indicating high discrimination. The item difficulty in the six boundaries in the 7-point Likert data are also acceptable.

Table s1 IRT parameters of the 36 items in SATS-36*

| **Item** | **b1** | **b2** | **b3** | **b4** | **b5** | **b6** | **Discrimination** |
| --- | --- | --- | --- | --- | --- | --- | --- |
| q1 | -4.148 | -3.924 | -3.344 | -2.493 | -1.733 | -0.866 | 1.452 |
| q2 | -4.118 | -3.876 | -3.377 | -2.577 | -1.573 | -0.605 | 1.532 |
| q3 | -2.944 | -2.541 | -1.971 | -0.806 | -0.016 | 0.654 | 2.171 |
| q4 | 3.358 | 1.777 | 0.423 | -1.075 | -2.515 | -4.176 | 0.678 |
| q5 | 3.558 | 2.134 | 1.883 | -2.816 | -1.853 | -2.475 | 0.393 |
| q6 | -3.895 | -2.476 | -0.785 | 1.064 | 2.974 | 4.696 | 0.719 |
| q7 | -1.003 | -1.892 | -2.562 | -3.312 | -3.837 | -4.617 | 1.297 |
| q8 | 11.885 | 7.013 | 6.427 | 5.506 | 0.493 | -5.832 | 0.425 |
| q9 | -3.608 | -3.172 | -2.292 | -1.367 | -0.540 | 0.351 | 1.828 |
| q10 | -3.531 | -2.901 | -2.311 | -1.374 | -0.582 | 0.309 | 1.886 |
| q11 | 0.473 | -0.516 | -1.479 | -2.393 | -3.142 | -3.869 | 1.261 |
| q12 | -2.739 | -2.003 | -1.278 | -0.159 | 0.776 | 1.650 | 1.619 |
| q13 | 0.597 | -0.620 | -1.446 | -2.480 | -3.250 | -4.119 | 1.038 |
| q14 | -4.012 | -3.538 | -2.893 | -1.954 | -1.013 | 0.055 | 1.485 |
| q15 | 2.974 | 1.547 | 0.506 | -0.943 | -2.166 | -3.224 | 0.786 |
| q16 | 0.890 | -0.181 | -1.006 | -2.185 | -2.954 | -3.725 | 1.326 |
| q17 | -3.377 | -2.473 | -1.687 | -0.547 | 0.492 | 1.440 | 1.307 |
| q18 | 3.246 | 1.533 | 0.614 | -0.584 | -2.190 | -3.895 | 0.756 |
| q19 | -2.749 | -2.142 | -1.572 | -0.460 | 0.209 | 1.051 | 2.649 |
| q20 | -2.637 | -2.135 | -1.526 | -0.607 | 0.058 | 0.935 | 3.072 |
| q21 | 1.488 | 0.041 | -1.072 | -2.245 | -3.403 | -4.436 | 1.069 |
| q22 | -4.755 | -1.867 | 0.319 | 2.924 | 4.760 | 7.358 | 0.475 |
| q23 | -2.692 | -2.040 | -1.484 | -0.510 | 0.295 | 1.214 | 2.572 |
| q24 | -5.945 | -5.027 | -4.153 | -2.437 | -0.855 | 0.939 | 0.858 |
| q25 | 0.430 | -0.601 | -1.543 | -2.761 | -3.498 | -4.249 | 1.193 |
| q26 | 6.398 | 3.870 | 2.186 | 0.277 | -1.881 | -4.341 | 0.515 |
| q27 | -4.799 | -4.448 | -3.834 | -2.934 | -2.443 | -1.556 | 1.147 |
| q28 | 1.240 | 0.220 | -0.439 | -1.286 | -2.261 | -3.089 | 1.290 |
| q29 | -2.601 | -2.083 | -1.485 | -0.616 | 0.042 | 0.787 | 2.696 |
| q30 | -14.914 | -11.094 | -7.489 | -3.922 | -0.709 | 3.084 | 0.337 |
| q31 | -3.130 | -2.506 | -2.019 | -0.995 | -0.173 | 0.751 | 2.363 |
| q32 | -3.745 | -2.773 | -1.985 | -0.637 | 0.439 | 1.814 | 1.402 |
| q33 | 0.279 | -0.777 | -1.643 | -2.576 | -3.137 | -3.780 | 1.404 |
| q34 | -7.570 | -6.518 | -4.679 | -2.603 | -0.678 | 1.356 | 0.630 |
| q35 | 3.676 | 1.789 | 0.538 | -1.013 | -2.484 | -3.740 | 0.764 |
| q36 | -8.075 | -6.195 | -4.532 | -1.947 | -0.070 | 1.734 | 0.633 |

*：b1-b6 are the item difficulties in the six boundaries in the 7-point Likert item.

The results item characteristic curve (ICC) of all the 36 items were shown as figure s1. The ICC analysis expressed the predicted probability of a certain response by the model. If the participants showed relatively low probabilities of all responses on a particular item, it suggests that the item was difficult to answer with low discrimination. We found that the items q5, q8 and q30 showed relatively low probabilities of all 7 responses, which corroborated a low discrimination as shown in table s1.


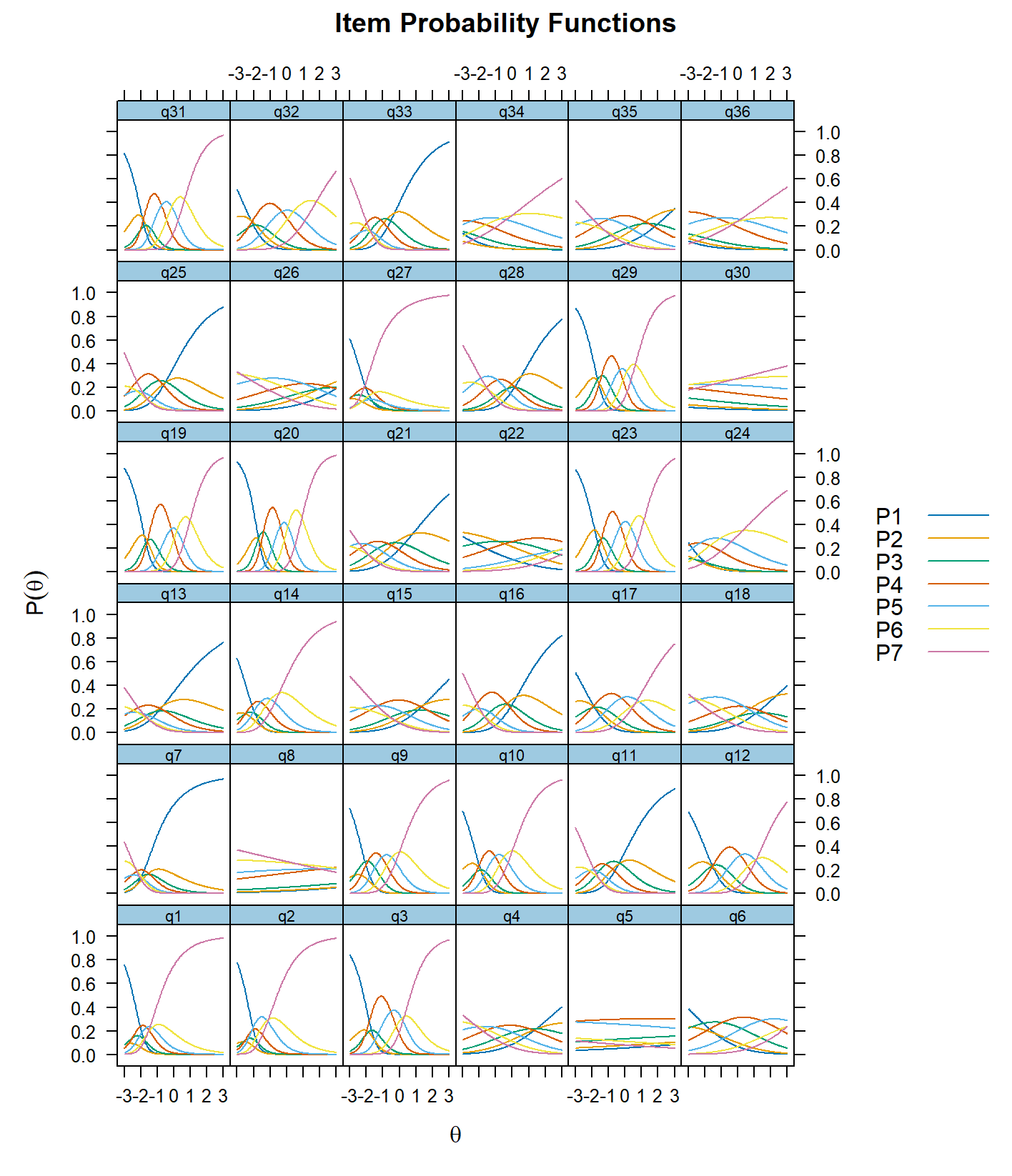


Figure s1 The item characteristic curve (ICC) of all the 36 items in SATS-36

The results of item information curve (IIC) were shown in figure s2, which was the indicator of measurement error for the items with higher values suggesting more contributions for the measure of the latent subscale.


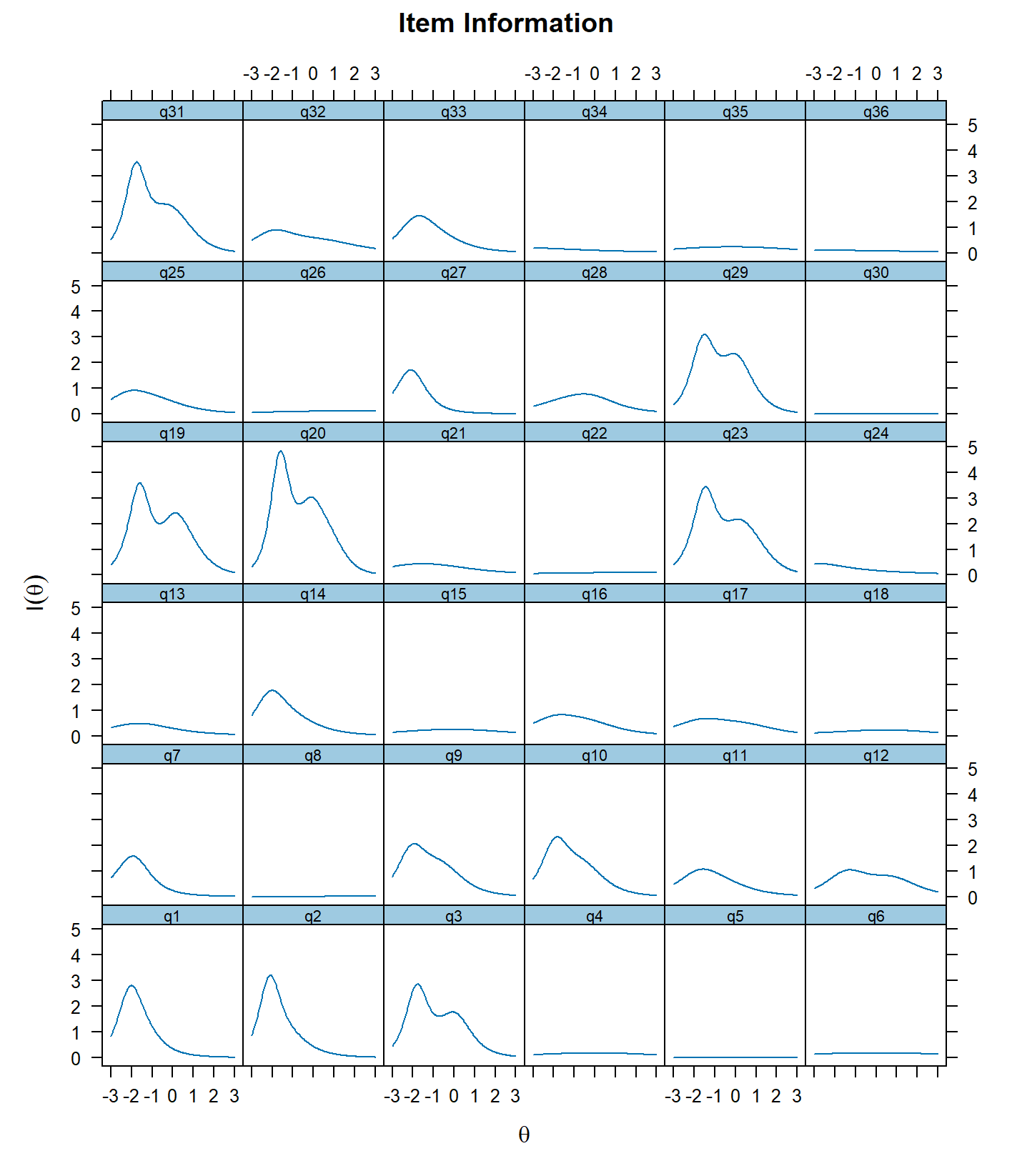


Figure s2 The item information curve (IIC) of all the 36 items in SATS-36

We also analyzed the reliability of the items as shown in figure s3. The empirical reliability and marginal reliability were calculated as 0.9486 and 0.9503, respectively, which was considered sufficiently to indicate the item reliability.


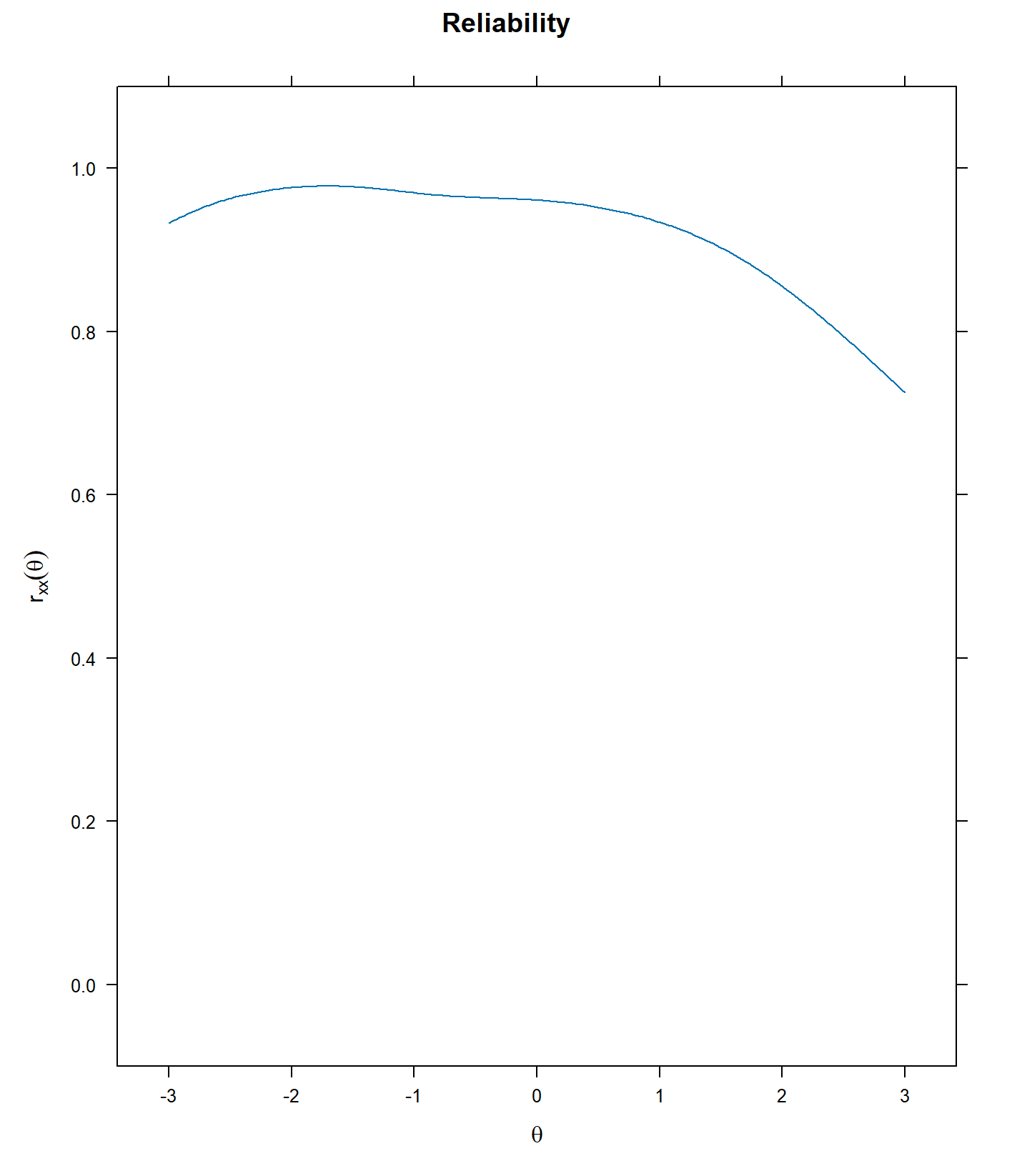


Figure s3 The reliability of all the 36 items in SATS-36

In summary, we considered IRT analysis treating 7-point Likert response as ordinary data showed an acceptable discrimination and reliability, which indicated similar accuracy and robustness of the results in the main manuscript.
